# Bacterial communities co-develop with respiratory immunity early in life, linking dysbiosis to systemic monocyte signature and wheezing

**DOI:** 10.1126/sciadv.adw1410

**Published:** 2025-10-17

**Authors:** Céline Pattaroni, Matthew Macowan, Roxanne Chatzis, Giulia Iacono, Bailey Cardwell, Mindy Gore, Adnan Custovic, Michael D. Shields, Ultan F. Power, Jonathan Grigg, Graham Roberts, Peter Ghazal, Jürgen Schwarze, Steve Turner, Andrew Bush, Sejal Saglani, Clare M. Lloyd, Benjamin J. Marsland

**Affiliations:** ^1^ Department of Immunology, School of Translational Medicine, Monash University, Melbourne, Australia.; ^2^ Imperial Centre for Paediatrics and Child Health, and National Heart and Lung Institute, Imperial College, London, UK.; ^3^ Wellcome-Wolfson Institute for Experimental Medicine, School of Medicine, Dentistry and Biomedical Sciences, Queen’s University Belfast, Belfast, UK.; ^4^ Centre for Child Health, Blizard Institute, Queen Mary University of London, London, UK.; ^5^ Human Development in Health School, University of Southampton Faculty of Medicine, Southampton, UK.; ^6^ NIHR Southampton Biomedical Research Centre, University Hospital Southampton NHS Foundation Trust, Southampton, UK.; ^7^ David Hide Asthma and Allergy Research Centre, St Mary’s Hospital, Newport, Isle of Wight, UK.; ^8^ School of Medicine, Systems Immunity Research Institute, Cardiff University, Cardiff, UK.; ^9^ Centre for Inflammation Research, Child Life and Health, The University of Edinburgh, Edinburgh, UK.; ^10^ Child Health, University of Aberdeen, Aberdeen, UK.; ^11^ NHS Grampian, Aberdeen, UK.; ^12^ Royal Brompton Hospital, London, UK.; ^13^ National Heart & Lung Institute, Imperial College, London. UK.

## Abstract

Early microbial colonization influences respiratory disease risk, yet mechanisms remain unclear. In a prospective birth cohort of 256 infants, we profiled bacterial, fungal, and viral communities in the upper airway and assessed local immune gene expression longitudinally and systemic gene expression at 1 year. Bacterial populations, not fungal or viral, correlated most strongly with immune development during the first 3 months, coinciding with composition shifts and immune-related gene expression changes, including interferon and adaptive immunity pathways. In contrast, the mycobiome and resident viruses showed no significant coevolution with host immunity. By 1 year, infants who previously wheezed displayed an upper airway microbiota enriched in *Haemophilus influenzae* and *Moraxella*, accompanied by a distinct local and systemic immune gene signature featuring elevated classical monocyte-related genes. These findings reveal a specific link between early-life bacterial dysbiosis, monocyte-related immunity, and wheezing onset, suggesting potential targets for early intervention in respiratory disease.

## Teaser

Bacteria-only airway microbiota comatures with newborn’s immunity, linking systemic monocyte signature with previous wheeze.

## INTRODUCTION

Birth marks the onset of microbial colonization of mucosal surfaces, including the respiratory tract, with the local mucosal microenvironment being the primary driver of microbial diversification (*1*). Host factors, along with environmental factors including delivery mode, breastfeeding, daycare, and vaccination lead to variation in the respiratory bacterial (*2*–*6*) and fungal microbiota (*7*) across respiratory niches. Multiple studies have reported substantial changes in the upper (*8*, *9*) and lower (*6*) airways’ microbiota within the initial days to weeks of life, followed by a stabilization thereafter. In addition to its gatekeeping function against respiratory pathogens, the respiratory microbiota is thought to play an important role in priming and shaping the local immune system during early life. This includes the establishment of immune tolerance (*10*) and mucosal barrier function through the regulation of immunoglobulin A (IgA)–mediated responses (*6*, *11*). While the effects of the bacterial microbiota on respiratory immune maturation have been increasingly investigated, the roles of viral and fungal airway colonization under steady-state conditions remain largely unknown.

Understanding host-microbe interactions is crucial, as early-life perturbations of the respiratory bacterial microbiota have been associated with the development of respiratory diseases. For example, the colonization of the upper airways with *Moraxella*, *Haemophilus*, or *Streptococcus* has been associated with subsequent wheezing and the development of asthma (*4*, *12*–*14*). Recurrent preschool wheezers frequently exhibit persistent bronchial bacterial infections involving the same pathogens, even during nonexacerbation periods (*15*–*17*). In addition, a strong connection exists between viral infections and bacterial dysbiosis involving these microbes, where disturbances in the bacterial community have been shown to either precede (*13*, *18*) or follow respiratory tract infections (*8*).

We hypothesized that dynamic host-microbial interactions within the upper airways shape local and systemic immune development during the critical first year of life, possibly influencing wheeze pathogenesis. To test this hypothesis, we performed multikingdom microbial and host transcriptomic profiling in two groups of participants from the Breathing Together cohort. The first was a longitudinal group (Long-Group) of children sampled at four different time points over the first year of life to investigate the development of host-microbial interactions in the upper airways. The second was a cross-sectional group (CS-Group) of children sampled at age one to ascertain host-microbial interactions in relation to prior wheezing during the first year of life. To our knowledge, this is the first observational study examining the dynamics of three microbial kingdoms, integrating both local (nasal) and systemic (blood) immune profiles, and exploring these interactions during healthy development and wheeze.

## RESULTS

### Characteristics of study populations

**Fig. 1.**
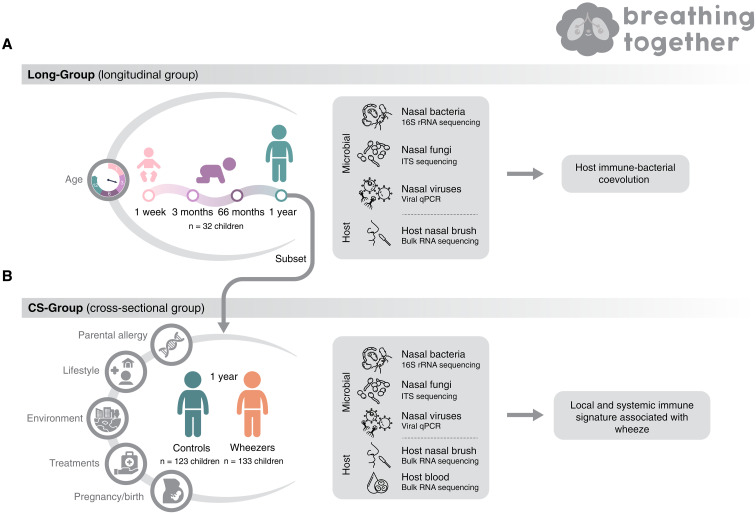
Long-Group and CS-Group from the Breathing Together birth cohort. (**A**) Samples from 32 children taken at four different time points in the first year of life constitute the Long-Group for which three microbial datasets (nasal bacterial taxonomic profile, nasal fungal taxonomic profile, and nasal viral qPCR profile) and one host dataset (nasal gene expression) were generated. (**B**) These children represent a subset of the CS-Group for which three microbial datasets (nasal bacterial taxonomic profile, nasal fungal taxonomic profile, and nasal viral qPCR profile) and two host datasets (nasal gene expression and blood gene expression) were generated from samples taken at 1 year of age for 256 children (123 healthy children and 133 children with parent-reported wheeze) alongside detailed metadata. Integration of these datasets revealed a shared local and systemic immune signature associated with wheeze.

Two groups of children from the Breathing Together study, which aims to identify the key epithelial and immune determinants of asthma in early life (*19*), were investigated. First, a Long-Group comprising 32 children with biological samples collected at four time points (1 week, 3 months, 6 months, and 1 year) to investigate host nasal (local) gene expression and microbial profiles (bacteria, fungi, and viruses) (Fig. 1A). Second, a CS-Group of 256 children at 1 year, who provided the same sample types as the Long-Group and in addition to blood for transcriptomic analysis (Fig. 1B). Of 256 children included in the cross-sectional analysis, 133 had parent-reported wheezing in the first year of life. Biological samples were obtained when wheezing symptoms were absent, in adherence to the established sampling protocol. Nasal microbiota profiling of both groups included bacterial 16*S* [*n* = 338 datasets after quality control (QC)] and fungal ITS (*n* = 212 datasets after QC) amplicon sequencing, paired with viral quantitative polymerase chain reaction (qPCR) profiling (*n* = 297 datasets after QC), to characterize the multiple components of the microbiota across the three major kingdoms. Host immune profiling was performed using RNA sequencing, focusing on local characterization (nasal brush *n* = 200 datasets after QC) for both CS-Group and Long-Group samples, and systemic analysis (whole blood *n* = 73 datasets after QC) for CS-Group samples only.

### Maturation of immunological profiles aligns with the development of bacterial but not fungal or viral communities during the first 12 months of life (Long-Group)

**Fig. 2.**
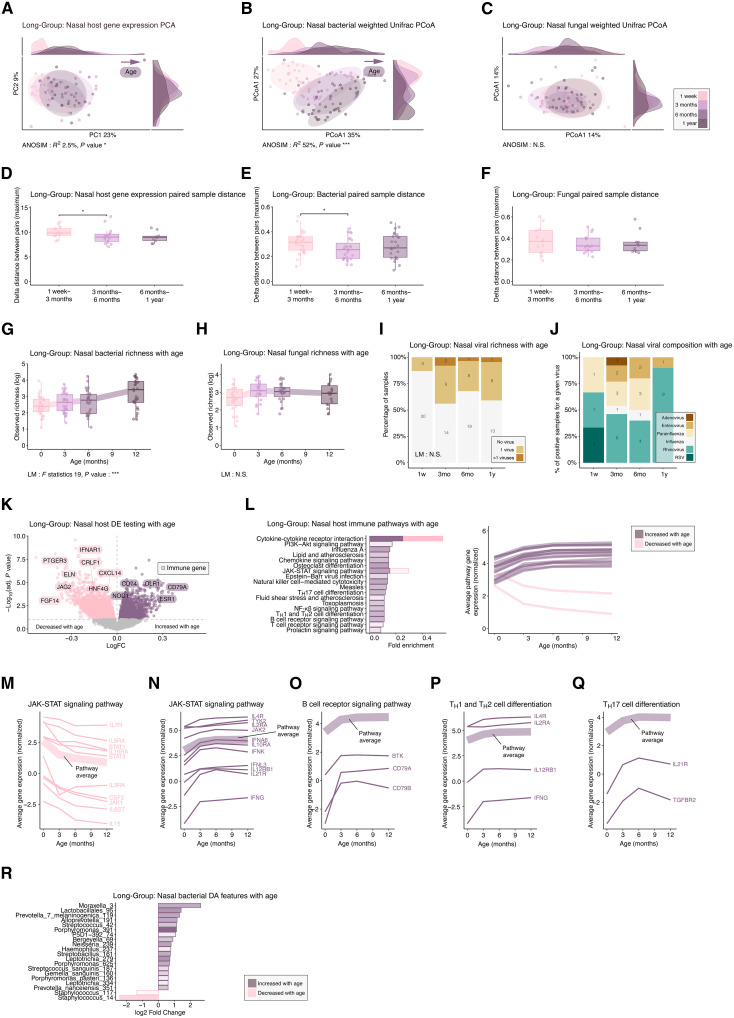
Nasal microbiota and host transcriptome show parallel development in the first year (Long-Group). (**A**) PCA of nasal transcriptomics across time points and density plots. (**B**) Principal Coordinate Analysis (PCoA) of bacterial and (**C**) fungal microbiota using UniFrac and density plots. (**D**) Transcriptome stability across three time points using maximum delta. (**E**) Bacterial and (**F**) fungal microbiota stability using UniFrac delta. (**G**) Bacterial and (**H**) fungal richness over time. (**I**) Respiratory viral detection per time point. (**J**) Virus composition in positive samples. (**K**) DE of nasal transcriptomics with age using limma, immune genes marked with squares. (**L**) KEGG pathway analysis of DE immune genes; top 20 pathways shown. Bar color indicates the direction of change in average gene expression with age treated as a continuous variable: Purple bars reflect pathways up-regulated with increasing age, and pink pathways down-regulated with age. Expression trends for selected genes and the pathway-level average, calculated as the mean normalized expression of all genes in each pathway at each time point; (**M**) JAK-STAT down with age. (**N**) JAK-STAT up with age. (**O**) B cell receptor signaling (**P**) T_H_1/T_H_2 and (**Q**) T_H_17 differentiation. (**R**) Differential abundance (DA) of nasal bacterial microbiota using LINDA between week 1 and year 1; top 20 taxa shown. Sample sizes: host: 1 week (*n* = 20), 3 months (*n* = 23), 6 months (*n* = 23), and 1 year (*n* = 15); bacteria: 1 week (*n* = 29), 3 months (*n* = 31), 6 months (*n* = 29), and 1 year (*n* = 28); fungi: 1 week (*n* = 22), 3 months (*n* = 21), 6 months (*n* = 23), and 1 year (*n* = 18); viruses: 1 week (*n* = 23), 3 months (*n* = 25), 6 months (*n* = 28), and 1 yr. (*n* = 22). Statistical tests: Permutational Multivariate Analysis of Variance [PERMANOVA (A to C)], Wilcoxon [(D) to (F)], linear model [(I) and (J)], limma (K), KEGG (L), and LINDA (R); FDR corrected for (K), (L), and (M). *P < 0.05 and **P < 0.001.

We first aimed to examine the development of the multikingdom microbiota alongside local host responses within the Long-Group. The principal components analysis (PCA) of host nasal transcriptomic data (Fig. 2A) and principal coordinate analysis of nasal bacteria (Fig. 2B) and fungi (Fig. 2C) revealed that both the overall host nasal gene expression and nasal bacterial composition were largely determined by age [analysis of similarities (ANOSIM) coefficient of determination (*R*^2^) = 3%, *P* value = 0.009 for host; *R*^2^ = 52%, *P* value < 0.001 for bacteria]. In contrast, fungal composition did not show significant age-related variation [*R*^2^ = 43%, *P* value = not significant (N.S.)]. A pronounced shift along the first axis was observed for both nasal host gene expression and bacterial profiles, with the most significant transition occurring between the first (1 week) and second (3 months) time points, a pattern not observed in the fungal data. To further address individual microbial changes over time, distances between consecutive sample pairs from the same individuals were compared. Host nasal gene expression (Fig. 2D) and bacterial (Fig. 2E) profiles were most unstable during the first two time intervals (1 week to 3 months Wilcoxon rank test *W* = 151, *P* value = 0.04 for host and *W* = 510, *P* value = 0.03 for bacteria). In contrast, distances between these time points were not different for fungi (1 week to 3 months Wilcoxon rank test *W* = 106, *P* value = N.S.) (Fig. 2F), suggesting that age is not a major driver of nasal fungal microbial variation for a given individual. Nasal bacterial richness significantly increased with age (linear model *F* statistic = 18.94, *P* value < 0.001) (Fig. 2G), whereas no such trend was observed for fungi (linear model *F* statistic = 1.72, *P* value = N.S.) (Fig. 2H). Viruses were detected in less than 50% of nasal samples at all time points and were particularly scarce in the first week of life (present in 3 of the 20 samples) (Fig. 2I). No age-related changes were linked to the presence of common respiratory viruses (linear model *F* statistic = 1.72, *P* value = 0.19), suggesting that, similar to fungi, steady-state nasal viral presence is age independent. In-depth analysis of viral composition highlighted that rhinovirus was the predominant virus detected in these asymptomatic children, present in 63% (20 of 32) of the virus-positive nasal samples (Fig. 2J). Last, we aimed to identify age-associated features through differential expression and abundance testing. Among all differentially expressed (DE) host nasal genes, 133 immune genes were up-regulated, while 121 immune genes were down-regulated, suggesting an age-associated immune switch (Fig. 2K). The pathway analysis of immune genes revealed several immune pathways that increased with age during the first year of life, with the most pronounced rise occurring between the first week and 3 months, followed by stabilization after 6 months (Fig. 2L and table S1). Further exploration of these gene groups revealed a shift within the Janus kinase (JAK)/signal transducers and activators of transcription (STAT) pathway in the first year of life. Genes showing decreased expression with age included those encoding key pathway members JAK1, STAT1, STAT3, interleukin-15 and its receptor (*IL15* and *IL15RA*), interleukin receptors (*IL6ST*, *IL7R*, *IL3RA*, and *IL5RA*), and granulocyte-macrophage colony-stimulating factor (*CSF2*) (Fig. 2M). Conversely, other components of the JAK/STAT pathway showed increased expression during the first year of life (Fig. 2N). These included JAK2 and TYK2 along with interferons (*IFNG*, *IFNK*, *IFNA6*, and *IFNL3*) and type I interferon receptor genes such as *IFNAR2*. Within the same pathway, γc cytokine family receptors (*IL2RA*, *IL21R*, and *IL4R*), alongside the structurally related interferon receptor gene *IL10RA* and *IL12RB1*, were also up-regulated. Pathways related to antiviral immunity also increased with age, which coincided with the increased interferon signals observed in the JAK/STAT pathway and also included downstream interferon regulatory element interferon regulatory factor 7 and nuclear factor κB inhibitor beta (*NFKBIB*) (table S1). Pathways central to adaptive immunity, particularly B cells and T cell responses, increased with age. This included the up-regulation of the B cell receptor signaling pathway (Fig. 2O), such as both subunits of the CD79 receptor Igα (*CD79A*) and Igβ (*CD79B*), alongside key downstream Bruton tyrosine kinase (*BTK*). T cell–related pathways [T helper 17 (T_H_17) cell differentiation/T_H_1 and T_H_2 cell differentiation] also increased, featuring key T_H_1 (*IFNG* and *IL12RB1*) (Fig. 2P), T_H_2 (*IL2RA* and *IL4R*), and T_H_17 (*TGFBR2* and *IL21R*) genes (Fig. 2Q). Furthermore, a pathway associated with natural killer (NK) cells (NK cell–mediated cytotoxicity), bridging innate and adaptive immunity, also showed an up-regulation in the first year of life, involving NK cell–specific genes such as killer cell lectin like receptor C1 (*KLRC1*) (table S1). Following the analysis of host gene expression, we next explored microbial features that exhibited differential abundance over the first year of life (Fig. 2R). Bacterial abundance changes included a reduction in *Staphylococcus* amplicon sequence variants (ASVs) (ASVs) and an increase in ASVs from common upper airway colonizers such as genera *Moraxella*, *Prevotella*, *Streptococcus*, *Porphyromonas*, *Neisseria*, and *Haemophilus*. In contrast to bacteria, neither fungal abundance nor viral presence showed a significant correlation with age. In summary, samples from the first year of life showed synchronized innate and adaptive immune cell maturation and development of the bacterial microbiota indicating a distinct age-associated pattern in host-bacterial interactions, independent of fungal or viral presence.

### Daycare and antibiotics affect bacterial and respiratory viruses at year 1 with a minimal impact on local host gene expression (CS-Group)

**Fig. 3.**
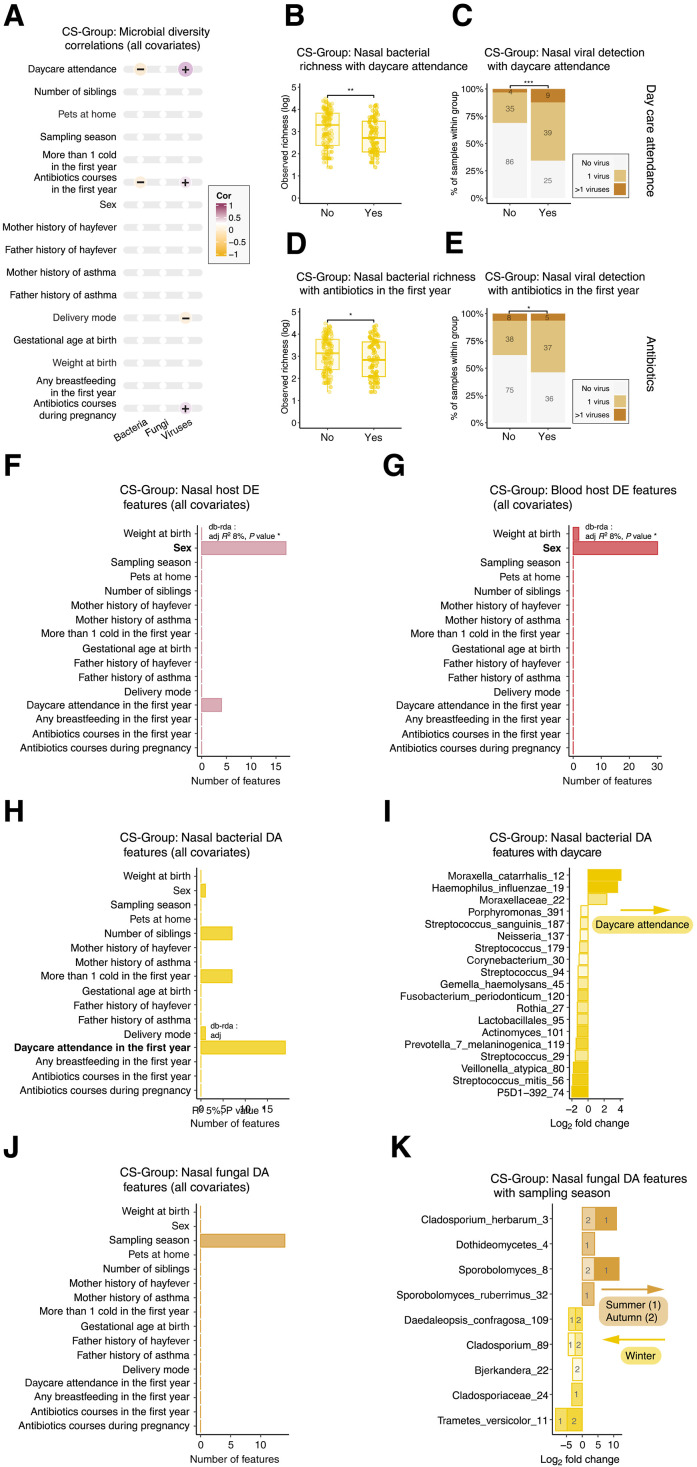
Environmental factors such as daycare and antibiotic treatment impact bacterial and viral components of the nasal microbiota (CS-Group). (**A**) Spearman correlations between nasal bacterial, fungal, and viral alpha diversity and covariates; positive (purple) and negative (yellow) associations represented, with color intensity and symbol size proportional to correlation strength. (**B**) Bacterial richness by daycare attendance. (**C**) Viral detection by daycare attendance. (**D**) Bacterial richness by antibiotic treatment in the first year. (**E**) Viral detection by antibiotic treatment in the first year. (**F**) DE genes in the nasal transcriptome per covariate using limma, with db-RDA for key variable selection. (**G**) DE genes in the blood transcriptome by covariate with db-RDA. (**H**) Differential abundance (DA) of nasal bacterial taxa by covariate using LINDA and db-RDA. (**I**) DA bacterial taxa associated with daycare, colored by significance level (adjusted *P* value). (**J**) DA fungal taxa by covariate using LINDA and db-RDA. Bar color intensity in DA plots proportional to statistical significance. (**K**) Fungal taxa associated with season (summer versus winter), colored by adjusted significance. Sample sizes: bacteria (*n* = 233), fungi (*n* = 153), viruses (*n* = 199), nasal host transcriptome (*n* = 122), and blood transcriptome (*n* = 73). Statistical methods: FDR-adjusted Spearman correlations (A), Wilcoxon tests [(B) and (D)], χ^2^ tests [(C) and (E)], limma and db-RDA [(F) to (H) and (J)], and LINDA (I and K); multiple testing correction applied to (A) and (F) to (K). *P < 0.05, **P < 0.01, and ***P < 0.001.

Integrating environmental and host factors is crucial to understand their role in shaping early life microbial interactions, as they can alter microbiota composition and potentially affect immune system development. To explore these interconnected factors, covariates collected from questionnaires at birth and 1 year were investigated in relation to both local (nasal) and systemic (blood) host gene expression, alongside the three sets of local microbial data at year 1 (CS-Group). Microbial richness and diversity of the three microbial kingdoms in correlation with the collected covariates were investigated first (Fig. 3A). Two factors had opposing impacts upon bacterial and viral richness. Daycare attendance emerged as the first influential factor, with children in daycare showing lower bacterial richness (*W* = 7830, *P* value < 0.01) (Fig. 3B), but a higher prevalence of viruses (*W* = 2907, *P* value < 0.001) (Fig. 3C). The use of antibiotics during the first year also followed this trend, with a decrease in bacterial richness (*W* = 7495, *P* value = 0.045) (Fig. 3D) and a rise in virus prevalence (*W* = 4025, *P* value = 0.046) (Fig. 3E). None of the factors investigated influenced fungal nasal richness. To further examine how these factors influence individual host and microbial traits, a distance-based redundancy analysis (rd-rda) was used, selecting variables of significance through model selection, combined with differential expression and abundance testing. Host data analysis identified sex as a key determinant of gene expression, responsible for 7 and 17% of the observed variation in nasal (Fig. 3F) and blood (Fig. 3G) samples, respectively. Daycare attendance was found to affect bacterial composition, accounting for 5% of the variance in the model (Fig. 3H). Subsequent differential abundance testing analysis showed increased ASV abundance of pathogenic bacteria such as *Moraxella* and *Haemophilus influenzae* in children attending daycare. Conversely, ASVs representing common healthy airway colonizers—such as *Prevotella*, *Veillonella*, and *Rothia*—were more abundant in children not attending daycare. The only factor that significantly affected fungal ASVs abundance was the season in which the nasal sample was taken (Fig. 3J). The majority of observed changes were associated with environmental fungi, with outdoor airborne molds such as *Cladosporium herbarum* (*20*) and members of the family *Sporobolomyces* showing higher abundance during summer and autumn, while *Trametes versicolor* was most abundant in winter months (Fig. 3K).

### Parent-reported wheeze associates with higher *H. influenzae* abundance and local expression of immunomodulatory genes at year 1 (CS-Group)

**Fig. 4.**
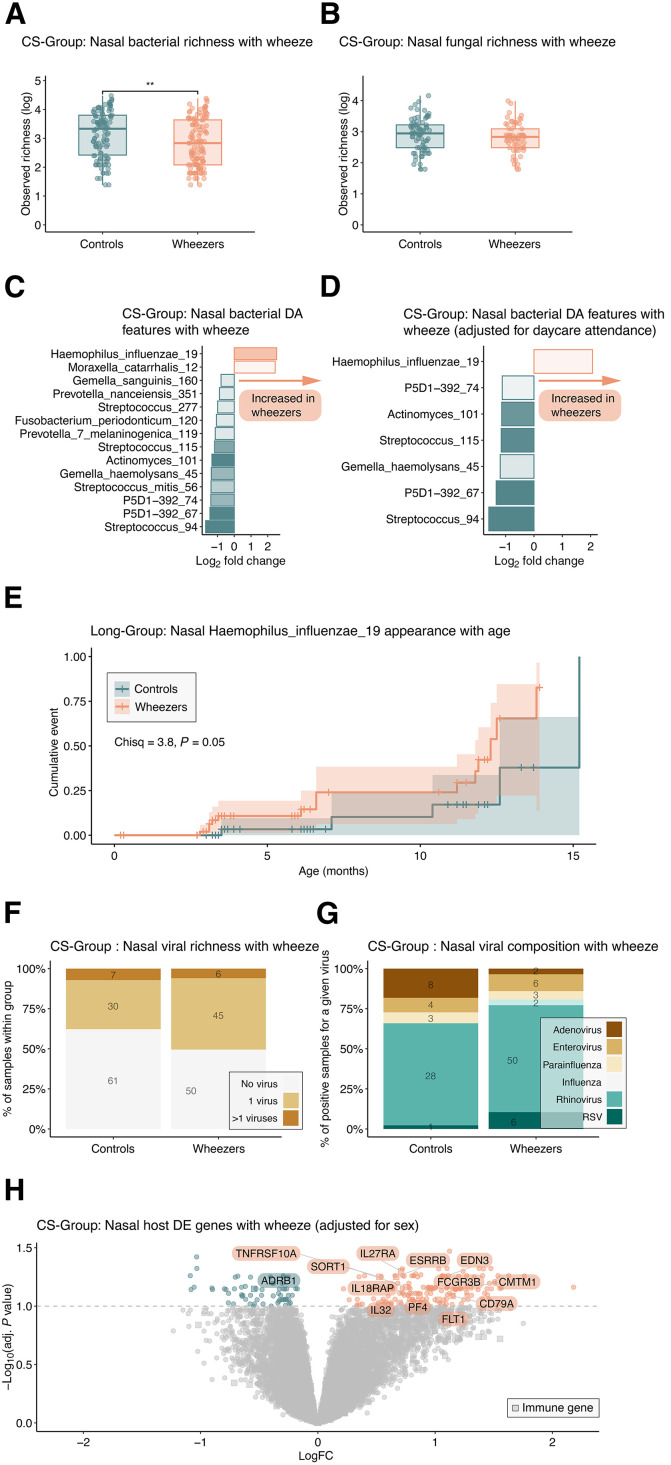
Parent-reported wheeze in the first year of life affects local nasal bacterial microbiota composition and host immune gene expression at steady state (CS-Group). (**A**) Observed bacterial and (**B**), fungal richness between parents-reported wheezers and controls. (**C**) Result of nasal bacteria LINDA testing between parent-reported wheezers and controls unadjusted and (**D**) adjusted for daycare attendance. (**E**) Kaplan-Meier curves representing the cumulative incidence of *H. influenzae 19* appearance (event) with age between parent-reported wheezers (orange) and controls (blue green) in the (Long-Group). (**F**) Nasal viral detection (no virus detected, 1 virus detected, more than 1 virus detected) and (**G**) detailed viral composition of virus-positive samples between parents-reported wheezers and controls. (**H**) Volcano plot depicting the result of limma DE testing using a sex-adjusted model comparing parents-reported wheezers and controls. Significantly DE genes (adjusted *P* value < 0.1) increased in wheezers (orange) or controls (blue green) with immune genes represented by a square. Sample sizes are wheezers, *n* = 119; controls *n* = 114 for bacteria, wheezers, *n* = 61; controls *n* = 77 for fungi, wheezers, *n* = 101; controls *n* = 98 for viruses, wheezers, *n* = 82; controls *n* = 40 for host nasal, wheezers, *n* = 46; controls *n* = 27 for host blood. Statistics represent the result of nonparametric Wilcoxon rank sum tests [(A) and (B)], χ^2^ tests [(C) and (D)], limma (E), LINDA [(F) and (G)], and log-rank test (H) with (multiple testing corrected for E to G) *P < 0.01.

We next investigated differences in host and microbial features for each individual omics dataset in parent-reported wheezers, children who had any wheezing events occurring within the first year, using samples collected at year 1 when they were asymptomatic (CS-Group). The age of onset for wheezing symptoms ranged from 3 to 12 months, with a median age of wheezing onset of 6 months. In assessing clinical covariates, the wheeze group showed significantly higher incidences of antibiotic use both during pregnancy (χ^2^ = 4.28, *P* value = 0.04) and in the first year of life (χ^2^ = 39.61, *P* value < 0.001), maternal history of asthma (χ^2^ = 8.11, *P* value = 0.004), pets (χ^2^ = 5.71, *P* value = 0.02), and more than one cold during the first year of life (χ^2^ = 54.96, *P* value <0.001) (table S2). The investigation of the nasal microbiota revealed a significant reduction in bacterial richness in wheezers (*W* = 8301, *P* value < 0.01) (Fig. 4A), with fungal richness remaining unchanged (*W* = 2625, *P* value = N.S.) (Fig. 4B). Differential abundance analysis, both without (Fig. 4C) and with (Fig. 4D) daycare correction, showed differences in taxa abundance including a notable increase in *H. influenzae* abundance in the wheezer group after adjustment. The presence of *Moraxella catarrhalis* was associated with previous wheezing only when daycare attendance was not factored in the model, suggesting that daycare attendance might influence or confound this relationship. Given the results observed in the CS-Group at 1 year, we subsequently investigated the longitudinal subset (Long-Group). We did not observe any significant differences in bacterial or fungal taxa between wheezers and controls at birth, 3 months, or 6 months. Similarly, no DE genes were detected at these early time points. Notably, the majority of wheezing cases in this cohort developed after the 6-month time point, limiting our ability to detect early microbial or immune differences. However, wheezers showed an earlier appearance of *H. influenzae*, as demonstrated by survival analysis (Chisq = 3.8, *P* value = 0.05) (Fig. 4E). Similar to fungi, there were no differences in the presence (Fig. 4F) or composition (Fig. 4G) of viruses between the two groups. Independent differential expression testing of host nasal genes unveiled 152 genes up-regulated and 63 genes down-regulated with wheeze with no changes detected in blood gene expression (Fig. 4H and table S3). Further examination of up-regulated immune genes revealed that receptors for both interleukin-18 (IL-18) and IL-27 cytokines [interleukin 18 receptor accessory protein (*IL18RAP*) and interleukin-27 receptor alpha genes (*IL27RA*), respectively] were up-regulated. Other up-regulated immune genes included the proinflammatory cytokine IL-32, the Fc fragment of IgG receptor IIIb (*FCGR3B*), involved in immune complex clearance, and CD79a (*CD79A*), the key component of the B cell receptor complex, among others. These changes in immune gene expression were moderate, with log fold changes (logFCs) not exceeding 1.15.

### Increased abundance of *H. influenzae* and *Moraxella* is associated with a local and systemic monocyte signature (CS-Group)

**Fig. 5.**
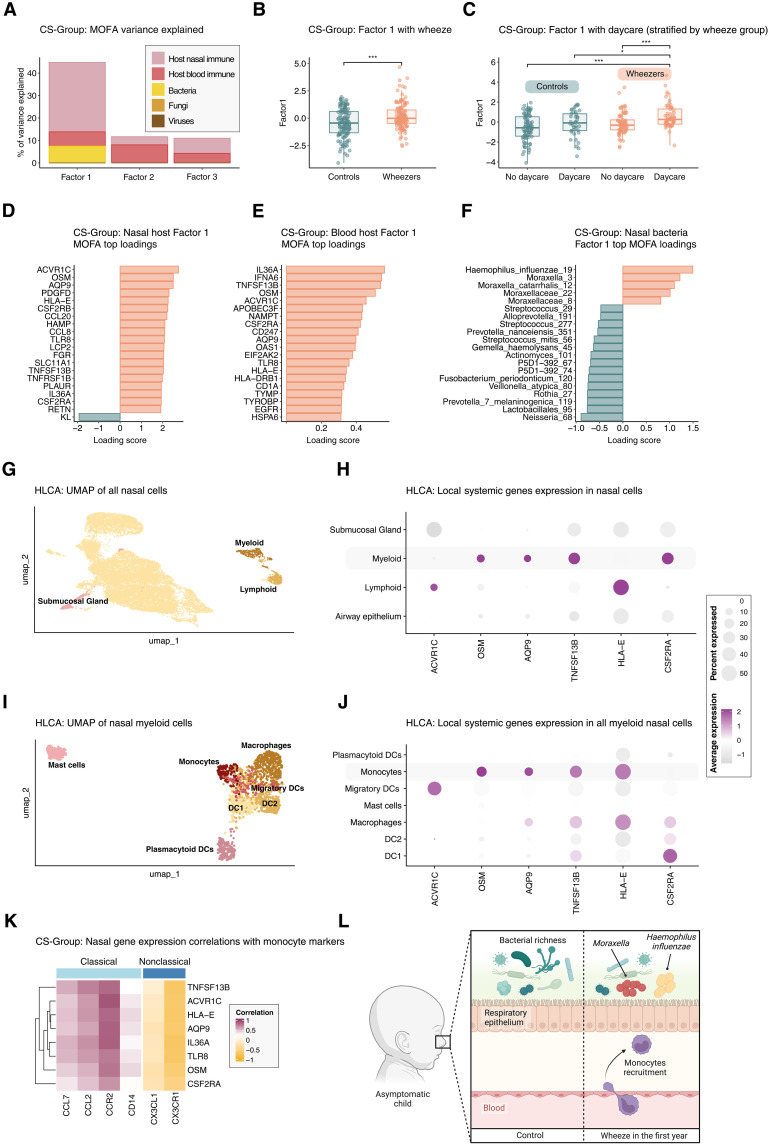
Multiomics data integration reveals a local and systemic monocyte immune signature associated with local bacterial increase of *Haemophilus* and *Moraxella* (CS-Group). (**A**) Percentage of variance explained by each latent factor of MOFA combining microbial (bacteria, fungi, and viruses) and host (nasal and systemic immune) datasets. (**B**) Factor1 values by parent-reported wheeze status. (**C**) Factor1 values by daycare attendance, stratified by wheezing. (**D**) Top 20 loading values of local (nasal) immune gene expression for factor 1. (**E**) Top 20 loading values of systemic (blood) immune gene expression for factor 1. (**F**) Top 20 loading values of local bacterial taxonomy for Factor1. (**G**) UMAP of nasal cells from the HCLA after batch correction and integration. (**H**) Dotplot of common local systemic genes identified by MOFA; dot size represents percentage of cells within a cell type, color intensity reflects average gene expression across cells (yellow = low, purple = high). (**I**) UMAP of the myeloid cell compartment from the HLCA nasal cell subset. (**J**) Dotplot of MOFA-identified local-systemic gene expression within the myeloid compartment. (**K**) Spearman correlation plot between local nasal expression of shared genes and monocyte markers. (**L**) Graphical summary of findings: nasal bacterial dysbiosis with increased *H. influenzae* and *Moraxella* sp. in wheezers associated with a local and systemic immune gene expression signature consistent with classical monocyte recruitment to the airways. Sample sizes: bacteria (*n* = 233), fungi (*n* = 153), viruses (*n* = 199), nasal host (*n* = 122), and blood host (*n* = 73). Statistical tests: Wilcoxon rank sum [(B) and (C)] and Spearman (K). *P < 0.05, and ***P < 0.001

Transitioning from independent host and microbial analyses of wheezers and controls, we next used a multiomics integrative approach using multiomics factor analysis (MOFA) to combine all data from the CS-Group in an unsupervised fashion. MOFA infers a low-dimensional representation of the data independently of group classifications, capturing key patterns of covariance across omics datasets through latent factors that represent the underlying principal axes of heterogeneity across the samples. This approach has the advantage of revealing interconnected features between host and microbial data while bypassing the subjectivity of parent-reported wheezing. The MOFA analysis of host immune nasal, host immune blood, bacterial, fungal, and viral data identified three latent factors, with factor 1 notably explaining 45% of the variance and integrating microbial (bacterial) data (7% of variance explained) with both nasal (31% of variance explained) and blood (6% of variance explained) host immune features (Fig. 5A). Factor 1 scores were higher in wheezers (*W* = 5904, *P* value < 0.001), underscoring a significant link between host-microbial features and wheeze despite the unsupervised nature of the analysis (Fig. 5B). Stratification based on daycare attendance within wheeze and control groups showed that factor 1 scores were significantly increased in wheezers attending daycare in comparison with other groups (Kruskal-Wallis χ^2^ = 27.54, *P* value < 0.001) (Fig. 5C). Inspection of factor 1’s top 20 features in both nasal (Fig. 5D) and blood host immune (Fig. 5E) datasets revealed that eght of these genes were expressed both locally and systemically (local systemic genes). Other factors loadings are presented in fig. S1. These included genes encoding for the activin receptor type-1C (*ACVR1C*, also known as *ALK7*), Oncostatin M (*OSM*), Aquaporin 9 (*AQP9*), tumor necrosis factor (TNF) superfamily member 13b (*TNFSF13B*, also known as *BAFF*), interleukin 36 alpha (*IL36A*), major histocompatibility complex class I E (*HLA-E*), Toll-like receptor 8 (TLR8), and colony stimulating factor 2 receptor subunit alpha (*CSF2RA*). Factor 1 covariation was characterized by increased abundance of *H. influenzae* and *Moraxella* genus ASVs, while ASVs such as *Neisseria*, *Prevotella melaninogenica*, and *Veillonella* were associated with lower factor 1 scores, partially mirroring the earlier independent differential analysis results (Fig. 5F). To investigate which cell types might underlie the observed local systemic gene signature, we mapped these genes onto single-cell transcriptomic data from the Human Lung Cell Atlas (HLCA) (see Materials and Methods) (Fig. 5G). The average expression of these genes was predominantly high in a small subset of myeloid cells, with the exception of *ACVR1C* and *HLA-E*, which showed lower expression levels (Fig. 5H). To further investigate the expression of local systemic genes in specific myeloid cell types within the nasal cavity under steady-state conditions, myeloid cells were then subsetted from the HLCA (Fig. 5I). We found that nasal monocytes predominantly expressed these local systemic genes, specifically *OSM*, *AQP9*, *TNFSF13B*, and *HLA-E*, while macrophages showed lower expression levels (Fig. 5J). Human peripheral blood monocytes are defined by their expression of markers including CD14, CD16, CD64, CCR2, and CX3CR1 (*21*). Classical monocytes are identified by the high expression of CD14 [lipopolysaccharide (LPS) co-receptor] and CCR2, a key mediator of monocyte migration, with relatively lower levels of CX3CR1 (fractalkine receptor), while nonclassical monocytes are marked by higher expression of CD16 (Fc gamma RIII) and CX3CR1. We also examined corresponding chemokine ligands such as CCL2, CCL7, and CX3CL1, important for monocyte recruitment and trafficking (*21*–*23*). Local systemic genes significantly correlated with classical monocyte markers *CD14* and *CCR2* gene expression, along with the expression of their corresponding ligands genes *CCL2* and *CCL7* [Spearman false discovery rate (FDR) < 0.05] (Fig. 5K). This pattern, along with the absence of positive correlations with nonclassical marker genes *CD16* and *CD64*–both of which were expressed at low levels and filtered out during preprocessing–and a negative correlation with the ligand-receptor pair genes *CX3CR1-CX3CL1*, indicates that these monocytes are of the classical type. In summary, the unsupervised multiomics integration of CS-Group samples revealed a unique local systemic host immune signature, aligning with a classical monocyte phenotype. This pattern was associated with bacterial profiles dominated by *H. influenzae* and *Moraxella*, suggestive of a generalized immune state that is sustained in wheezers even when nonsymptomatic (Fig. 5L).

## DISCUSSION

We aimed to address the relationship between host-microbial interactions and the development of early respiratory immunity, with a focus on wheezing during the first year of life. The analysis of the longitudinal cohort (Long-Group) provided insights into the development of host-microbial interactions in infants’ healthy upper airways from birth to 1 year. We observed pronounced changes in the bacterial microbiota, particularly between birth and 3 months of age. This period marked an increase in typical upper airways colonizers (*24*), such as *Prevotella*, *Dolosigranulum*, *Streptococcus*, *Porphyromonas*, including pathobionts of the *Moraxella* and *Haemophilus* genera, and a decline in species from the *Staphylococcus* genus, aligning with bacterial microbiota trajectories described in other studies (*4*–*6*, *8*, *18*, *25*). Notably, we found that these shifts in bacterial composition coincided with significant changes in local gene expression. Among the most dynamically regulated immune pathways was JAK/STAT signaling, which showed a coordinated transcriptional shift in the first months of life. Early-expressed genes such as *STAT1*, *STAT3*, *JAK1*, *IL15*, *IL15RA*, and cytokine receptors including *IL6ST*, *CSF2*, and *IL5RA* declined with age. These were progressively replaced by increased expression of *JAK2*, *TYK2*, multiple interferons (IFNs) (*IFNG*, *IFNA6*, and *IFNL3*), the interferon receptor *IFNAR2*, and γc cytokine receptors (*IL2RA*, *IL21R*, *IL4R*, and *IL10RA*). This pattern aligns with findings that neonatal macrophages exhibit exaggerated IL-6–induced STAT3 phosphorylation and heightened acute-phase responses due to low SOCS3 expression, a key negative regulator, which may explain the early dominance and later suppression of STAT3-related signaling (*26*). Conversely, the delayed up-regulation of interferon pathway genes reflects the known deficiency of type I IFN responses in neonates, driven by reduced numbers and function of plasmacytoid dendritic cells (*27*–*29*) and align with earlier research conducted in a similar population, demonstrating that gene expression modules related to interferon production were not only connected to early viral infections in infants but also exhibited a distinct increase with age (*8*). Together, these findings indicate a developmental “switch” in airway cytokine signaling networks potentially orchestrated by microbial colonization during this immunologically formative period.

We also noted a developmental shift toward adaptive immunity, including B cell receptor signaling and T_H_1/T_H_2/T_H_17 cell differentiation. While our current understanding of the development of adaptive immunity in the airways is limited, evidence from the gastrointestinal field has highlighted the essential role of the microbiota in its induction. For example, indications that T_H_17 differentiation might be influenced by the microbiota emerged from observations that intestinal T_H_17 cells in mice are not detectable until around 3 to 4 weeks of age (*30*). In the gastrointestinal tract, T_H_17 cell development is hindered without microbiota (*30*), with segmented filamentous bacteria promoting T_H_17 maturation (*31*), B cell activation (*32*–*34*), and IgA production, which regulate microbial composition and immune balance (*35*, *36*). In a murine model, a single aspiration of a blend of human oral commensals (*Streptococcus mitis*, *Veillonella parvula*, and *P. melaninogenica*) was able to induce a T_H_17 response in the lower airway, which effectively reduced vulnerability to subsequent *Streptococcus pneumoniae* infection (*37*). Moreover, axenic- and antibiotic-treated mice exhibit impaired T_H_17 responses in the airways, which has been linked to the airway microbiota (*38*). The distinct bacterial-host immune coevolution we have observed suggests that similar mechanisms might be operational in the airways, particularly during the critical first three months, a potential “window of opportunity” (*39*) for the development of the immune system.

A distinctive aspect of this study is the investigation of the mycobiome and respiratory viruses at steady state in a longitudinal pediatric cohort. Contrary to our initial hypothesis, we observed no coevolution between host immunity and nonbacterial microbial kingdoms in the longitudinal group and, similarly, no evidence of interaction in the cross-sectional group at year 1. Although the airway mycobiome, particularly lower airways fungi such as *Aspergillus*, has been linked to several chronic diseases including asthma, cystic fibrosis, and chronic obstructive pulmonary diseases (*40*), its role in establishing baseline immune homeostasis remains elusive, even in the gut (*41*). On the other hand, multiple longitudinal cohort studies have established a connection between early respiratory microbiota development, notably the presence of *Haemophilus* and *Moraxella* species, and susceptibility to viral respiratory tract infections (*4*, *8*, *18*, *42*). In our study, the absence of an association between the presence of respiratory viruses and host immune gene expression in both groups suggests that residual viruses, in the absence of symptoms, may not directly influence the immune system. However, we did observe a correlation between previous wheezing and the presence of *H. influenzae* and *Moraxella*. Given the established link between these bacteria and acute respiratory infections, we cannot dismiss the possibility that acute viral infections might affect the bacterial microbiota or influence susceptibility to viral infections, which were not examined in the current study. We also investigated how various factors affect the microbiota and immune system at year 1 (CS-Group). We found that host gene expression was unaffected by factors other than sex. Daycare attendance significantly influenced the bacterial microbiota and viral presence; antibiotics showed a similar trend, consistent with findings from other studies (*1*, *4*, *18*, *43*). Only seasonal changes affected fungal composition in the upper airways, suggesting that the steady-state mycobiome in the upper airways essentially reflects the fungi present in the air. This is in line with reported high abundance of *Sporobolomyces* in UK residential air samples, which shows a distinct seasonal pattern (*44*).

Building on our initial findings, we aimed to determine whether these early life host-microbial interactions were associated with parental reported wheezing in the first year of life. Standard differential expression and abundance analyses revealed moderate host local immune gene expression differences between groups and variations in bacterial abundances, also linked with daycare. However, the most important findings emerged from our integrative multiomics approach, which combined multikingdom microbial data with host immune gene expression, both nasal and systemic. We identified a multiomics factor capturing the covariation between specific bacterial features and immune genes, suggesting potential host-microbial interactions. This multiomics factor was significantly increased in wheezers and even more so in individuals attending daycare. The top bacterial contributors to this factor included the pathobionts *H. influenzae* and *Moraxella*, contrasting with reduced abundance of typical healthy airway colonizers such as *Neisseria*, *Prevotella*, and *Veillonella*. When examining the genes explaining this factor in both local and systemic datasets, the same key genes—including *ACVR1C*, *OSM*, *AQP9*, *TNFSF13B* (*BAFF*), *IL36A*, *HLA-E*, *TLR8*, and *CSF2RA*—emerged as top features in both sets. These findings suggest a shared immune transcriptional program present across both mucosal and systemic compartments. To understand the cellular origin of this immune signature, we mapped these genes to nasal single-cell transcriptomic data from the HCLA. This revealed predominant expression in myeloid cells, particularly nasal monocytes, offering a plausible explanation for the presence of these genes in both nasal and blood compartments, consistent with the migratory nature of circulating monocytes. Some genes also showed lower-level expression in macrophages, suggesting a broader innate immune signature and a potential multi-pronged response. While HLA-E showed more specific enrichment in the lymphoid compartment, it was not a major focus of our cell-type interpretation, as the majority of local-systemic genes were most highly expressed in monocytes. Correlation with canonical monocyte markers (CD14 and CCR2) and their chemokine ligands (CCL2 and CCL7) further supports monocytes as the most likely contributors to this local systemic immune signal. Together, our findings support a biologically plausible hypothesis: airway dysbiosis involving LPS-rich Gram-negative organisms such as *H. influenzae* and *Moraxella* may lead to sustained recruitment and activation of monocytes.

Supporting this hypothesis, increased levels of OSM have been reported in asthma (*45*, *46*), and recent findings identified OSM as the most significant predicted upstream regulator of the transcriptional response in patients with severe asthma (*47*). The same study demonstrated in vivo that OSM is necessary and sufficient to drive pathophysiological features observed in severe asthma following exposure to LPS or *Klebsiella pneumoniae* (*47*). In addition, single-cell RNA sequencing from human lung biopsies revealed macrophages as the dominant cell-type expressing OSM (*47*). Building on this, this study found that LPS strongly induces OSM in human monocyte-derived macrophages (MDMs) and murine bone marrow–derived macrophages (BMDMs), underscoring the critical role of bacterial triggers in OSM-mediated airway inflammation (*47*). AQP9 has been shown to be involved in immune functions such as longevity of memory T cells (*48*) and both neutrophil and macrophage migration (*49*, *50*). Microbial products including LPS are known to activate B cells, either directly or indirectly, for example, through the induction of BAFF from myeloid cells (*51*–*53*). Furthermore, BAFF protein concentration was increased in nasal lavage samples from infants with Rous sarcoma virus (RSV)–associated bronchiolitis, which was directly correlated with *H. influenzae* abundance (*54*). The inhalation of LPS in healthy volunteers leads to monocytes recruitment in the lungs within 8 hours (*55*), and in mice, LPS treatment led to a significant increase in CCR2-dependent alveolar monocytes, whereas mice lacking CCR2 showed reduced monocyte recruitment (*56*). Together, these observations support a model in which airway colonization by *H. influenzae* and *Moraxella* promotes a low-grade, monocyte-driven inflammatory state via LPS-mediated immune activation. However, the temporal dynamics of this relationship remain unclear. It is possible that bacterial dysbiosis precedes and contributes to wheezing or, alternatively, that prior viral infections or inflammatory events create a niche that favors colonization by these taxa. This ambiguity is consistent with previous studies showing that bacterial shifts involving *H. influenzae* and *Moraxella* can both precede (*13*, *18*) and follow viral infections (*8*).

As with all observational cohort studies, our findings are correlational and not designed to establish causality. Nonetheless, the integration of longitudinal microbiota data with paired local and systemic transcriptomics provides a framework for generating mechanistic hypotheses. A key insight from our analysis is the potential recruitment of circulating monocytes in response to airway dysbiosis. While direct quantification of monocyte levels was not feasible due to the limited blood volumes obtainable from 1-year-old children, we inferred the most likely cellular origin of the gene signature using the HCLA. Although derived from adult tissue, it remains the best available reference for respiratory immune cells and allowed us to infer likely cell types in the absence of pediatric single-cell datasets. Another limitation is that wheezing status was based on parental report, which introduces subjectivity and lacks clinical validation. However, the core multiomic findings identified through unsupervised factor analysis were entirely independent of wheeze classification. This data-driven, label-free strategy greatly reduces the risk of bias introduced by grouping misclassification. The identification of *H. influenzae* and *Moraxella* as key microbial features aligns with prior studies linking these taxa to wheeze and asthma risk (*4*, *12*, *14*), further supporting the biological relevance and generalizability of our results. In contrast, the monocyte-associated transcriptional signature identified in our analysis is previously unreported and warrants further validation. To further define the biological meaning of these results, future studies should incorporate longitudinal sampling before, during, and after wheezing episodes and long-term clinical follow-up. These efforts will be essential to clarify causality and determine whether the microbial-immune interactions identified here represent viable targets for early intervention in wheeze-prone children.

In conclusion, our study reveals a unique coevolution between bacteria and the host immune system in the first year of life, distinct from fungi or viruses, with a notable shift in the first three months. Children who wheezed in their first year showed a dysbiotic airway microbiota, dominated by *H. influenzae* and *Moraxella*. This dysbiosis was linked to a generalized immune gene expression signature in both blood and nasal samples, driven by circulating classical monocytes. These findings shed light on the early-life interplay between microbiota and the immune system, highlighting how bacterial imbalances can shape systemic and local immune landscapes and affect respiratory health.

## MATERIALS AND METHODS

### Ethics approval and consent to participate

Informed parental consent was obtained before inclusion in the study, and ethical approval was obtained from the Regional Ethics Committee (REC reference: 16/LO/1518).

### Breathing Together cohort

This study includes children from the Breathing Together birth cohort (*19*), with recruitment activities conducted across five centres in the United Kingdom: Aberdeen, Edinburgh, Imperial College London, Queen Mary University London, and Isle of Wight, spanning from February 2017 to April 2019. Breathing Together cohort inclusion criteria were a gestational age of over 37 weeks and the provision of written parental consent. The exclusion criteria encompassed multiple pregnancy, detection of maternal group B *Streptococcus* (via vaginal swab or urine culture), the necessity for continuous positive airway pressure (CPAP) or ventilatory support, major congenital disorders (examples include congenital heart disease and cystic fibrosis), and anticipated challenges in follow-up (such as planned relocation). For this study, exclusions also extended to instances where either host or microbial samples were unavailable or if data on early-life wheezing were missing. The classification of participants into wheezers and controls was established on the basis of parental responses to a questionnaire given when the child reached 1 year of age. Children were classified as wheezers if there were any reported instances of wheezing during their first year. The control group consisted of those children whose parents reported no wheezing, no treatments for respiratory issues, absence of nocturnal dry cough, and no diagnosis of bronchiolitis within the same period.

### Participants sampling

Nasal swabs were collected using eSwabs (COPAN Diagnostics) and interdental brushes (Dentocare 620, 2.7 mm in diameter) for microbiota and host cell sampling, respectively. For blood collection, 50 μl of whole blood was obtained via capillary puncture using the Minivette POCT system (Sarstedt). Nasal brushes content was released in RLT lysis buffer (QIAGEN) with 2-mercaptoethanol (Sigma-Aldrich), and all samples were stored at −80°C with an addition of RLT lysis buffer (QIAGEN) with 2-mercaptoethanol (Sigma-Aldrich) for host cells.

### Bacterial 16*S* and fungal ITS amplicons sequencing

ESwab media were centrifuged at 14,000*g* for 10 min at 4°C. To enhance fungal DNA retrieval, pellets were treated with 300 U of lyticase (Sigma-Aldrich) at 37°C for 30 min with gentle shaking (500 rpm). Lysates were further processed using the DNeasy UltraClean Microbial Kit (QIAGEN) according to the manufacturer’s protocol, and DNA was eluted in 40 μl of microbial DNA-free water (QIAGEN), following the manufacturer’s guidelines in a controlled environment (laminar flow hood decontaminated with deoxyribonuclease solution and ultraviolet-treated) to avoid microbial DNA contamination. Negative controls included eSwab (opening and closing of a tube at the different sampling sites), extraction (microbial DNA-free water processed through the kit), and PCR (PCR reaction with microbial DNA-free water instead of DNA template). Each sample was amplified in two different reactions, the first one with custom barcoded primers targeting the bacterial 16*S* rDNA v1-v2 region (F-27/R-338) and the second one with custom barcoded primers targeting fungal internal transcribed spacer region 1 (ITS1) region. Primers used were 16*S* (forward: 5′AATGATACGGCGACCACCGAGATCTACACTATGGTAATTCCAGMGTTYGATYMTGGCTCAG-3′; reverse: 5′-CAAGCAGAAGACGGCATACGAG-ATACGAGACTGATTNNNNNNNNNNNNAAGCTGCCTCCCGTAGGAGT-3′) and ITS (forward: 5′-AATGATACGGCGACCA-CCGAGATCTACACGGCTTGGTCATTTAGAGGAAGTAA-3′; reverse:5′-CAAGCAGAAGACGGCATACGAGATNNNNNNNNNNNNCGGCTGCGTTCTTCATCGATGC-3′), where the N sequences represent the sample-specific 12-nucleotides Golean barcodes. PCR reaction consisted of microbial DNA-free water, 1 μl of each primer at 5 μM, 2.5 μl of Accuprime PCR buffer II (Thermo Fisher Scientific), 10 μl of DNA template, and 1 μl of Accuprime Taq polymerase (Thermo Fisher Scientific) with initial denaturation 3 min at 94°C, followed by 35 cycles (16*S*) or 40 cycles (ITS) of 30-s denaturation at 94°C, 30-s annealing at 56°C (16*S*) or 52°C (ITS), and 60-s elongation at 68°C, with a final extension at 68°C for 10 min. Amplicons were quantified using a Fragment Analyzer (Agilent Technologies) with the High Sensitivity NGS Fragment Analysis kit, pooled at equimolar amounts and purified using AMPure XP bead cleanup system (Beckman Coulter). Denatured library pools were sequenced on a MiSeq platform with a MiSeq Reagent Kit v2 (500 cycles).

### Viral qPCR

Extracted RNA and DNA were processed for qPCR using the Bosphore SARS-CoV-2/Respiratory Pathogens Panel Kit v1 (Anatolia Geneworks) according to the manufacturer’s protocol on a QuantStudio 6 platform (Thermo Fisher Scientific). Given the lack of a universal viral amplicon target, multiplex qPCR offers a sensitive and scalable method for detecting the major wheeze-associated viruses in children (*57*) (RSV, human rhinovirus, human metapneumovirus (MPV), and influenza), all of which are covered by this panel. This kit allows the detection of 13 viruses [severe acute respiratory syndrome coronavirus 2 (SARS-CoV-2, RSV A&B, influenza A&B, enterovirus, MPV, adenovirus, human parainfluenza 1/2/3/4, and rhinovirus. A Ct threshold of <40 was applied, corresponding to fluorescence levels above baseline noise and within the exponential phase of amplification. This threshold was consistently used across all samples, and negative controls showed no amplification below this value.

### Host RNA sequencing

RNA was isolated from cell lysates using the Quick-RNA Microprep Kit (Zymo Research), following the instructions provided in the manufacturer’s guide. Library preparation was performed using the NEBNextUltra Directional RNA Library Prep Kit for Illumina (New England Biolabs), and resulting libraries were sequenced on an Illumina NovaSeq platform with a Reagent kit S4 (150 cycles).

### Amplicons sequencing data preprocessing

Processing of the raw sequencing data was executed through the microbiome-dada2 workflow, as outlined in the code availability section, using the dada2 (*58*) R package (version 1.22.0). Raw FastQ files underwent demultiplexing via the iu-demultiplex function (version 2.7) of the illumina-utils (*59*) toolkit. This was followed by the removal of primers and adapters using cutadapt (*60*) (version 2.10), and the subsequent steps included filtering and trimming of reads, creation of sequencing error models, dereplication of sequences, inference of ASVs, merging of paired-ends, and removal of chimeric sequences. Taxonomy assignment for bacterial 16*S* ASVs was performed using both the SILVA database (*61*) train set and species assignment dataset (version 138.1) to ensure precise sequence matching. Similarly, fungal ITS ASVs were taxonomically classified using the UNITE (*62*) database’s general release as of May 2021. A phylogenetic tree of ASV sequences was constructed through multiple alignment processes using the DECIPHER (*63*) R package (version 2.22.0), followed by the creation of a neighbor-joining tree with the phangor (*64*) R package (version 2.8.1). This tree served as the basis for a maximum likelihood tree analysis using the generalized time-reversible model with Gamma rate variation (GTR + G + I), as previously detailed in our references. The identification of contaminant taxa was carried out using the decontam (*65*) R package (version 1.20.0) with a prevalence method at a threshold of 0.3 and contaminant features were removed prior to further filtering (fig. S2). All ASV tables with corresponding tree and taxonomy were imported in the phyloseq (*58*) R package (version 1.44.0). ASVs with less than 5% prevalence or unclassified at the phylum level were excluded, and samples yielding fewer than 5000 ASVs were also removed. Closely related ASVs were agglomerated using single-linkage clustering with the tip_glom function from the phyloseq package (*h* = 0.05). The Shannon index and observed richness were calculated using the estimate_richness function of phyloseq. Last, ASV counts underwent normalization using cumulative sum scaling via the calcNormFactors function of the MetagenomeSeq (*66*) R package (version 1.42.0) with log transformation for beta diversity analysis.

### Host sequencing data preprocessing

Raw FastQ files were processed using the nf-core/rnaseq pipeline (version 3.10.1) of the nf-core collection of workflows (*67*) executed with Nexflow (*67*) (version 22.10.4). Briefly, reads were aligned to the GRCh38 reference genome with STAR (*68*) (version 2.7.10a) and GRCh38.104 annotation file. Transcript quantification was performed using Salmon (*69*) (version 1.9.0) and length-scaled genes count used for downstream analyses were generated from the transcript level abundances using tximport (*70*) R package (version 1.12.0). Samples with less than 10 Mio mapped reads were excluded, and genes with low expression were removed using the filterByExpr function from the edgeR (*71*) R package (version 3.42.4) with default parameters. Sequencing batch effects for blood samples were removed using the removeBatchEffect function of limma R package (version 3.56.2). Only protein coding genes were retained for downstream analysis, and immune genes were determined using the InnateDB (*72*) retrieved in April 2023. Gene counts were transformed to logCPM using the voom function from the limma (*73*) R package (version 3.56.2).

### Microbial differential abundance testing, analysis of similarities, and distance-based redundancy analysis

Microbial differential abundance testing was performed using LinDA (*74*), a method specifically developed for microbiome compositional data, implemented in the microeco (*75*) R package (version 0.20.0) at the ASV level. LinDA applies log-ratio transformation to handle relative abundance data appropriately and supports multivariable linear modeling, enabling adjustment for confounding factors. Models were defined as “~Parameter1 + Parameter2” for more linear modeling with more than one variable. ANOSIM was performed on the weighted Unifrac distance matrix using the adonis2 function from the vegan R package (version 2.6.4). To evaluate the impact of covariates on microbiota beta diversity and identify the most relevant variables, we performed distance-based redundancy analysis (dbRDA) using the dbrda function of R vegan package (version 2.6.4). dbRDA was performed on the weighted Unifrac distance matrix, followed by stepwise variable selection guided by *R*^2^ values using the ordi2step function (vegan package version 2.6.4), and resulting *P* values were FDR-corrected using the p.adjust function from the stats R package (version 4.3.1).

### Host differential abundance testing and pathway analysis

Voom-transformed gene counts were used for differential expression testing using the limma R package (version 3.56.2). A linear model was constructed as follows “~Parameter1 + Parameter2” and used to fit gene expression data, and empirical Bayes statistics were applied to determine DE genes. Benjamini-Hochberg (BH) procedure was used for adjusting *P* values. Kyoto Encyclopedia of Genes and Genomes (KEGG) pathway analysis of immune genes was executed separately for up-regulated and down-regulated immune genes using the enrichKEGG function from the clusterProfiler (*76*) R package (version 4.8.2). The enrichment significance was assessed using hypergeometric testing and BH adjusted for multiple comparisons, and only pathways with a minimum DE gene counts ≥ 4 were reported.

### Intra-individual microbiota and gene expression stability

Intra-individual stability of microbial data was assessed by calculating the weighted Unifrac distance between two consecutive timepoints for children with a minimum of 3 complete samples set out of 4 timepoints. For gene expression data, stability was assessed by calculating the maximum distance between two consecutive time points for children with a minimum of three complete timepoints.

### Multiomics data integration

Multiomics integration was conducted using MOFA2 (*77*) (version 1.10.0) incorporating four distinct matrices. Before integration, the bacterial and fungal matrices underwent a 10% prevalence threshold filter to the bacterial and fungal matrices, followed by centered log-ratio transformation with the transform function of the microbiome R package (version 1.22.0). Voom-normalized host nasal and blood gene expression datasets were refined to exclusively encompass immune genes. Data and model training options were set as default with the number of factors set to 3.

### HCLA analysis

The core integrated HLCA was downloaded and imported into a Seurat object using the Seurat (*78*) R package (version 4.3.0.1). Only annotated nasal cells were retained. Nasal single-cell data from the different studies were then split and normalized using SCTransform (“v2”) from the sctransform (*79*) R package (version 0.3.5) and variable features were identified with FindVariableFeatures. Batch correction for studies was performed by selecting 2000 variable features using SelectIntegrationFeatures. Integration anchors were determined using FindIntegrationAnchors with rpca reduction (k.anchor = 50). The final integration—incorporating these anchors and additional local systemic genes TNFSF13B, AQP9, OSM, ACVR1C—was performed using the IntegrateData function. UMAP (Uniform Manifold Approximation and Projection) was used for dimensionality reduction using 20 PCA dimensions. Myeloid cells were subset and underwent neighbor finding and clustering before characterization using HCLA labels and manual annotation. The expression of the four genes of interest was assessed using the DotPlot function. Local systemic positive Spearman gene expression correlations with monocytes markers were performed on the voom-normalized gene counts using the corr.test function from the psych R package (version 2.3.6) with FDR correction for multiple testing using the p.adjust function from the stats R package (version 4.3.1).

### Statistical analyses

Statistical analyses were conducted as outlined in Materials and Methods. For comparisons involving two or more groups, the Wilcoxon rank sum exact test was employed using the wilcox.test function from the stats R package (version 4.3.1). To allow maximal reproducibility in functions requiring random pseudo-numbers, a global fixed random seed number was set to 2.
