# Supplementary material for: Bacterial communities co-develop with respiratory immunity early in life, linking dysbiosis to systemic monocyte signature and wheezing
Source: Sci Adv. 2025 Oct 17;11(42):eadw1410. doi: 10.1126/sciadv.adw1410 (PMC12533588; doi:10.1126/sciadv.adw1410)
Supplement: Supplementary file 1 — Figs. S1 and S2 Tables S1 to S3 [file sciadv.adw1410_sm.pdf]

Science Advances
AAAS

Supplementary Materials for

Bacterial communities co-develop with respiratory immunity early in life, linking dysbiosis to systemic monocyte signature and wheezing

Céline Pattaroni *et al.*

Corresponding author: Céline Pattaroni, celine.pattaroni@monash.edu

Sci. Adv. **11**, eadw1410 (2025)
DOI: 10.1126/sciadv.adw1410

This PDF file includes:

Figs. S1 and S2
Tables S1 to S3

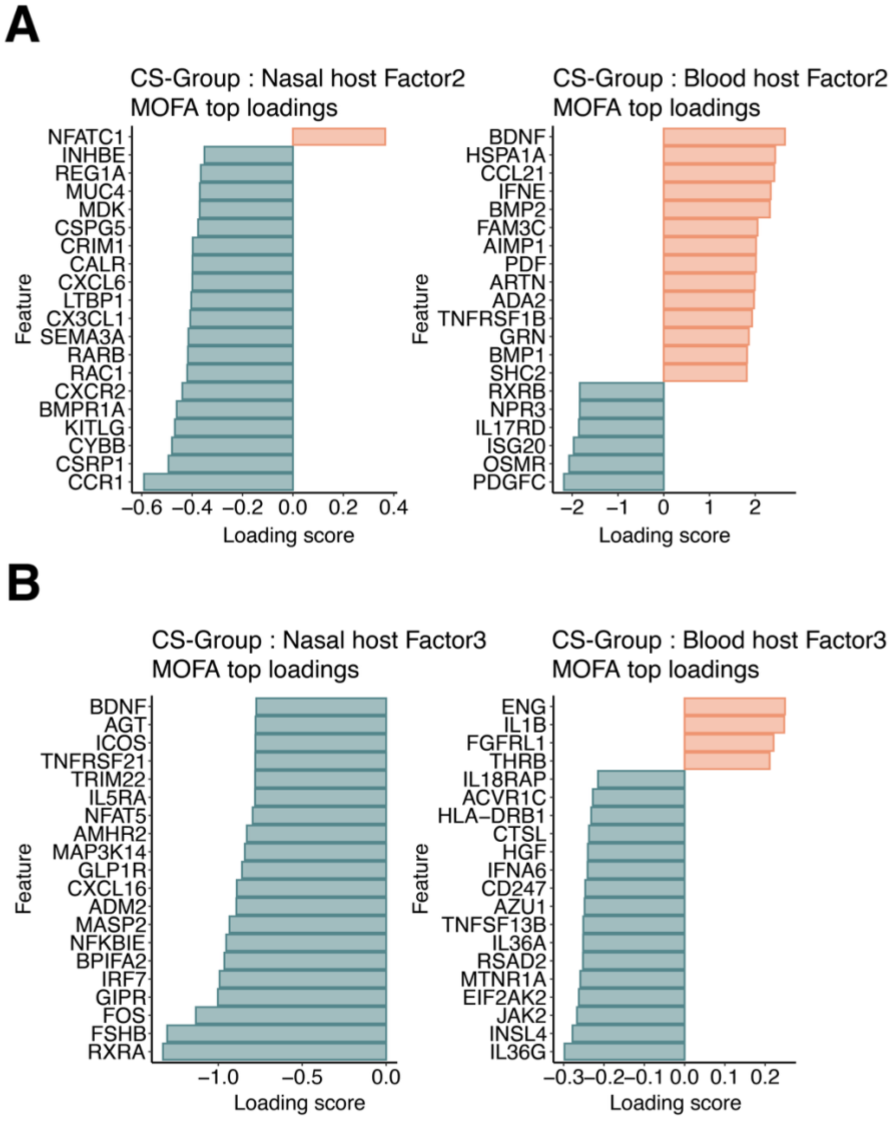

Fig. S1. Multi-omics Factor Analysis (MOFA) top loadings for Factor2 and Factor3.

(**A**) Top 20 loading values of local nasal (left panel) and blood systemic (right panel) immune gene expression for Factor2. (**B**) Top 20 loading values of local nasal (left panel) and blood systemic (right panel) immune gene expression for Factor3.

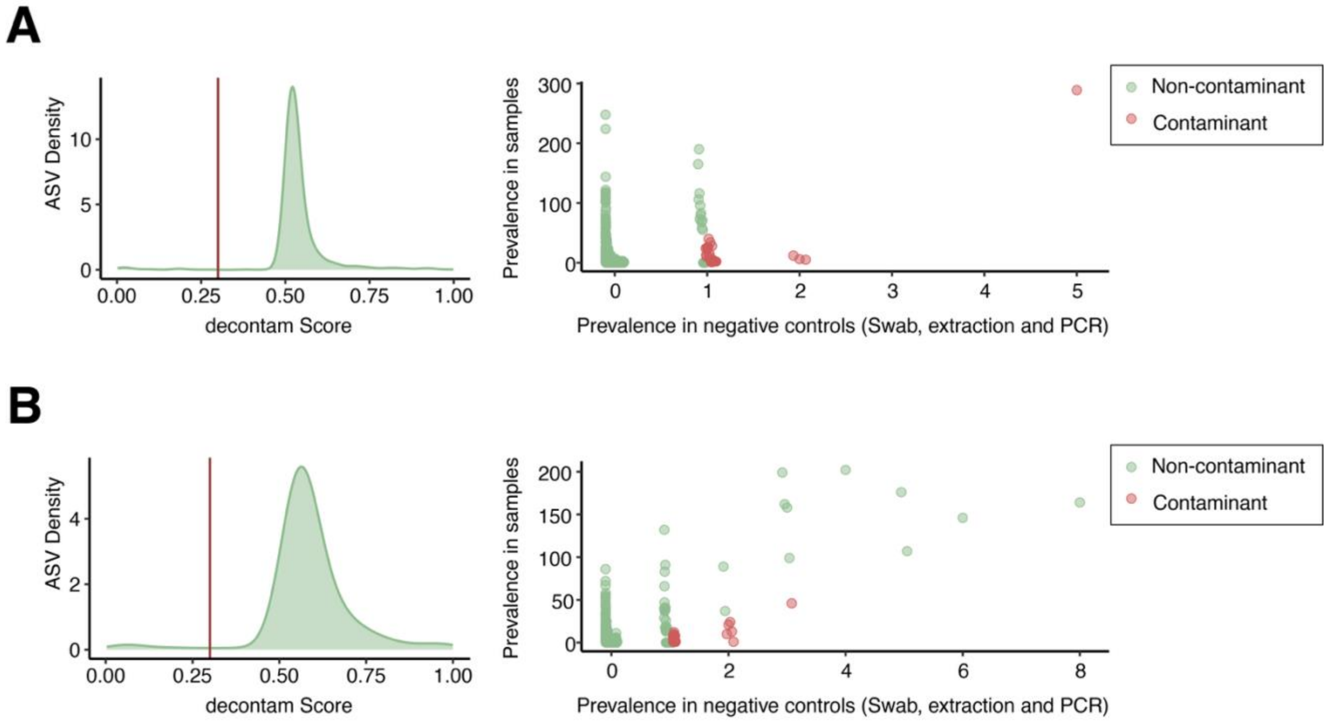

Fig. S2. Identification and removal of contaminant taxa.

(**A**) Decontam results for bacterial (16S rRNA) amplicon sequencing data. The left panel shows the distribution of ASV scores calculated using the prevalence method (threshold = 0.3). The right panel displays the prevalence of each ASV in true samples versus negative controls (eSwab, extraction, and PCR blanks); contaminants tend to show higher prevalence in negative controls relative to true samples. (**B**) Corresponding decontam output for fungal (ITS) amplicon data, showing ASV score distribution (left) and prevalence plot (right). Contaminant ASVs identified by this method were removed prior to downstream microbial analyses.

Description	GeneRatio	P.Value	adj.P.Val	Genes
Cytokine-cytokine receptor interaction (down with age)	29/97	2.87E-20	5.43E-18	IFNAR1/CXCL14/EPOR/GDF15/INHBB/TGFB3/CSF2/IL1RL2/IL15/IL6ST/IL7R/TNFSF11/XCL2/CCR4/INHBC/IL3RA/CXCL17/CCL28/IL15RA/TNFRSF8/BMP5/NGF/LEPR/TNFRSF12A/GDF3/CXCR1/FASLG/IL5RA/BMPR1A
Cytokine-cytokine receptor interaction (up with age)	30/117	8.06E-19	1.68E-16	IL1R2/CD40/IFNK/TNFRSF21/GHR/TNFRSF11A/IL17D/IL17RC/IFNG/IL2RA/IFNAR2/TNFRSF10A/IL21R/LTBR/IL4R/CCR9/IL16/IL1A/CCL21/IFNA6/IL12RB1/TGFBR2/TNFSF13B/BMP2/IL10RA/IL37/IFNL3/IL32/CXCL3/TNFSF8
Osteoclast differentiation	17/117	3.61E-12	3.76E-10	FCGR3B/AKT1/RELA/PIK3CB/TNFRSF11A/IFNG/IFNAR2/CYLD/IKBKB/MAP3K14/IL1A/NFKB1/TYK2/TGFBR2/PPP3CA/BTK/LCP2
JAK-STAT signaling pathway (up with age)	18/117	5.40E-12	3.76E-10	AKT1/IFNK/GHR/JAK2/PIK3CB/PDGFA/IFNG/IL2RA/IFNAR2/IL21R/IL4R/SOS2/IFNA6/TYK2/IL12RB1/PTPN6/IL10RA/IFNL3
Influenza A	18/117	8.09E-12	4.22E-10	HLA-DRA/AKT1/RELA/JAK2/PIK3CB/IRF7/IFNG/IFNAR2/TNFRSF10A/IKBKB/OAS1/ADAR/IL1A/NFKB1/IFNA6/NFKBIB/TYK2/ICAM1
Natural killer cell mediated cytotoxicity	16/117	1.92E-11	8.01E-10	FCGR3B/KLRC1/SHC1/RAC2/PIK3CB/SH3BP2/SHC2/IFNG/IFNAR2/TNFRSF10A/SOS2/IFNA6/PTPN6/PPP3CA/ICAM1/LCP2
Th17 cell differentiation	14/117	1.12E-10	3.90E-09	HLA-DRA/RELA/JAK2/IFNG/IL2RA/IL21R/IKBKB/IL4R/NFKB1/NFKBIB/TYK2/IL12RB1/TGFBR2/PPP3CA
B cell receptor signaling pathway	13/117	1.55E-10	4.63E-09	CD79A/RAC2/AKT1/RELA/PIK3CB/IKBKB/SOS2/NFKB1/NFKBIB/PTPN6/CD79B/PPP3CA/BTK

Th1 and Th2 cell differentiation	13/117	1.79E-10	4.67E-09	HLA-DRA/RELA/JAK2/IFNG/IL2RA/IKBKB/IL4R/NFKB1/NFKBIB/TYK2/IL12RB1/PPP3CA/JAG1
Measles	15/117	3.30E-10	7.66E-09	AKT1/RELA/PIK3CB/IRF7/IL2RA/IFNAR2/IKBKB/OAS1/ADAR/IL1A/NFKB1/IFNA6/NFKBIB/TYK2/HSPA6
Chemokine signaling pathway	17/117	5.02E-10	1.05E-08	ARRB1/PTK2/SHC1/RAC2/AKT1/RELA/JAK2/PIK3CB/PIK3CG/SHC2/IKBKB/CCR9/SOS2/CCL21/NFKB1/NFKBIB/CXCL3
NF-kappa B signaling pathway	13/117	9.68E-10	1.84E-08	CD40/RELA/TNFRSF11A/CYLD/IKBKB/LTBR/MAP3K14/CCL21/NFKB1/TNFSF13B/CXCL3/ICAM1/BTK
Toxoplasmosis	13/117	1.95E-09	3.39E-08	HLA-DRA/AKT1/CD40/RELA/JAK2/PIK3CG/IFNG/IKBKB/NFKB1/NFKBIB/TYK2/IL10RA/HSPA6
Lipid and atherosclerosis	17/117	2.87E-09	4.61E-08	OLR1/AGER/PTK2/AKT1/CD40/RELA/JAK2/PIK3CB/IRF7/TNFRSF10A/IKBKB/NFKB1/IFNA6/PPP3CA/CXCL3/ICAM1/HSPA6
Epstein-Barr virus infection	16/117	8.56E-09	1.28E-07	HLA-DRA/AKT1/CD40/RELA/PIK3CB/IRF7/IFNAR2/IKBKB/OAS1/MAP3K14/NFKB1/IFNA6/NFKBIB/TYK2/ICAM1/BTK
JAK-STAT signaling pathway (down with age)	14/97	3.28E-09	3.10E-07	IFNAR1/EPOR/JAK1/STAT3/CSF2/IL15/IL6ST/IL7R/IL3RA/SOS1/IL15RA/LEPR/IL5RA/STAT1
Prolactin signaling pathway	10/117	2.60E-08	3.62E-07	ESR1/SHC1/AKT1/RELA/JAK2/PIK3CB/TNFRSF11A/SHC2/SOS2/NFKB1

Fluid shear stress and atherosclerosis	13/117	3.68E-08	4.81E-07	PTK2/RAC2/AKT1/IL1R2/RELA/PIK3CB/PDGFA/IFNG/IKBKB/IL1A/NFKB1/HMOX1/ICAM1
PI3K-Akt signaling pathway	20/117	4.57E-08	5.61E-07	PTK2/AKT1/RELA/GHR/JAK2/PIK3CB/PIK3CG/PDGFA/IL2RA/IFNAR2/IKBKB/FGF16/IL4R/SOS2/NFKB1/IFNA6/FGFR4/GDNF/INSR/MET
T cell receptor signaling pathway	12/117	6.18E-08	7.18E-07	AKT1/RELA/PIK3CB/IFNG/IKBKB/SOS2/MAP3K14/NFKB1/NFKBIB/PTPN6/PPP3CA/LCP2

Table S1.

Long-Group immune pathway analysis results showing the top 20 pathways changing with age in the first year of life.

Covariate	Controls, *n* = 123	Wheezers, *n* = 133	P.Value
**Daycare attendance**			0.055
No	84 / 122 (69%)	75 / 133 (56%)	
Yes	38 / 122 (31%)	58 / 133 (44%)	
Data not available	1		
**Siblings**			0.084
No	65 / 122 (53%)	55 / 132 (42%)	
Yes	57 / 122 (47%)	77 / 132 (58%)	
Data not available	1	1	
**Pets**			0.017*
No	88 / 123 (72%)	75 / 133 (56%)	
Yes	35 / 123 (28%)	58 / 133 (44%)	
**Sampling season**			0.076
autumn	37 / 123 (30%)	43 / 133 (32%)	
spring	17 / 123 (14%)	33 / 133 (25%)	
summer	42 / 123 (34%)	31 / 133 (23%)	
winter	27 / 123 (22%)	26 / 133 (20%)	
**Country**			0.908
England	74 / 123 (60%)	82 / 133 (62%)	
Scotland	49 / 123 (40%)	51 / 133 (38%)	
**Cold during the first year**			<0.001***
1 or less colds in the first year	52 / 123 (42%)	4 / 132 (3.0%)	
More than 1 colds in the first year	71 / 123 (58%)	128 / 132 (97%)	
Data not available		1	
**Antibiotics during the first year**			<0.001***
No	100 / 123 (81%)	56 / 133 (42%)	
Yes	23 / 123 (19%)	77 / 133 (58%)	
**Sex**			0.109
Female	66 / 123 (54%)	57 / 133 (43%)	
Male	57 / 123 (46%)	76 / 133 (57%)	
**Mother history of hayfever**			0.313
No	80 / 122 (66%)	78 / 133 (59%)	
Yes	42 / 122 (34%)	55 / 133 (41%)	
Data not available	1		
**Father history of hayfever**			0.551
No	87 / 121 (72%)	90 / 133 (68%)	
Yes	34 / 121 (28%)	43 / 133 (32%)	
Data not available	2		
**Mother history of asthma**			0.004**
No	104 / 121 (86%)	92 / 131 (70%)	
Yes	17 / 121 (14%)	39 / 131 (30%)	
Data not available	2	2	
**Father history of asthma**			1.00
No	87 / 119 (73%)	95 / 131 (73%)	

Yes	32 / 119 (27%)	36 / 131 (27%)	
Data not available	4	2	
**Delivery mode**			0.358
C-section	35 / 123 (28%)	46 / 133 (35%)	
Vaginal	88 / 123 (72%)	87 / 133 (65%)	
**Gestational age at birth**			0.953
38 weeks or below	55 / 123 (45%)	61 / 133 (46%)	
above 40 weeks	68 / 123 (55%)	72 / 133 (54%)	
**Weight at birth**			0.177
Weight at birth < 3.5kg	81 / 123 (66%)	75 / 132 (57%)	
Weight at birth > 3.5kg	42 / 123 (34%)	57 / 132 (43%)	
Data not available		1	
**Antibiotics during pregnancy**			0.039*
No	102 / 123 (83%)	94 / 132 (71%)	
Yes	21 / 123 (17%)	38 / 132 (29%)	
Data not available		1	

Table S2.

CS-Group participants characteristics.

Gene	logFC	AveExpr	t	P.Value	adj.P.Val
AK5	1.12	0.48	4.96	2.32E-06	3.38E-02
HEATR4	-1.03	1.05	-4.77	5.19E-06	3.78E-02
JAK3	1.48	3.88	4.58	1.14E-05	4.75E-02
SH2D2A	1.10	1.37	4.52	1.48E-05	4.75E-02
C2orf66	1.38	-0.47	4.45	1.90E-05	4.75E-02
ABHD14B	-1.03	0.68	-4.45	1.96E-05	4.75E-02
ZNF454	1.41	0.09	4.38	2.50E-05	4.78E-02
SLC37A1	1.03	0.62	4.34	2.94E-05	4.78E-02
TAP2	0.72	4.99	4.34	2.95E-05	4.78E-02
IL27RA	0.71	3.02	4.30	3.47E-05	5.02E-02
HOXC13	1.24	-0.13	4.28	3.79E-05	5.02E-02
DNAJC5B	0.83	2.25	4.24	4.35E-05	5.28E-02
AKNA	0.88	5.98	4.22	4.80E-05	5.38E-02
AFF2	1.40	-1.03	4.18	5.44E-05	5.48E-02
SLC36A2	-1.09	-1.41	-4.15	6.15E-05	5.48E-02
KIF3C	0.66	2.14	4.12	7.07E-05	5.48E-02
P2RX5	1.63	-1.41	4.10	7.37E-05	5.48E-02
EPB41L3	0.80	3.69	4.10	7.39E-05	5.48E-02
SSUH2	-0.29	6.62	-4.10	7.57E-05	5.48E-02
LAGE3	0.40	2.91	4.09	7.70E-05	5.48E-02
CD200	1.11	-1.55	4.08	8.08E-05	5.48E-02
ACTR3	1.06	2.15	4.06	8.89E-05	5.48E-02
ASB2	0.82	1.95	4.05	8.99E-05	5.48E-02
FLAD1	1.54	1.13	4.05	9.15E-05	5.48E-02
CYP7A1	0.72	3.53	4.04	9.47E-05	5.48E-02
INA	0.82	3.96	4.03	9.91E-05	5.48E-02
CLVS2	1.27	0.87	4.02	1.02E-04	5.48E-02
TMEM144	-0.68	5.35	-3.99	1.13E-04	5.52E-02
ZNF652	1.76	2.00	3.99	1.14E-04	5.52E-02
TMEM8B	0.74	1.47	3.99	1.14E-04	5.52E-02
MTRF1	1.20	-1.31	3.98	1.18E-04	5.52E-02
ESRRB	0.67	2.03	3.96	1.25E-04	5.61E-02
CAB39L	-0.40	6.01	-3.96	1.29E-04	5.61E-02
EDN3	1.08	-0.02	3.95	1.31E-04	5.61E-02
POU2F2	1.05	2.66	3.92	1.48E-04	5.73E-02
RASAL3	0.85	2.92	3.91	1.52E-04	5.73E-02
RAB2A	-0.25	7.67	-3.91	1.53E-04	5.73E-02
SH3BP4	0.35	4.55	3.90	1.57E-04	5.73E-02
METTL8	-0.29	5.48	-3.90	1.57E-04	5.73E-02
NRBF2	1.33	1.35	3.89	1.62E-04	5.73E-02
TNFRSF10A	0.53	3.77	3.89	1.63E-04	5.73E-02
SLC37A2	0.54	5.54	3.89	1.65E-04	5.73E-02
BDKRB1	-0.70	1.43	-3.88	1.71E-04	5.76E-02
RASSF9	-0.57	12.22	-3.87	1.74E-04	5.76E-02

TBC1D31	1.51	2.38	3.84	1.94E-04	6.29E-02
UMODL1	0.68	3.97	3.82	2.08E-04	6.49E-02
RWDD2B	1.46	1.47	3.81	2.19E-04	6.49E-02
HPDL	1.51	-2.16	3.81	2.22E-04	6.49E-02
PPP1R16B	0.78	3.79	3.80	2.26E-04	6.49E-02
GDE1	-0.38	7.25	-3.80	2.26E-04	6.49E-02
DRC1	-0.31	6.12	-3.80	2.31E-04	6.49E-02
TPD52	-0.38	6.79	-3.80	2.32E-04	6.49E-02
UGT2A3	-0.62	6.84	-3.77	2.50E-04	6.88E-02
MCTP1	0.90	3.31	3.75	2.74E-04	6.90E-02
CMTM1	1.30	-1.46	3.75	2.76E-04	6.90E-02
BAIAP2	0.95	-1.68	3.75	2.76E-04	6.90E-02
TMEM9	0.78	3.82	3.74	2.77E-04	6.90E-02
FGD6	-0.65	3.09	-3.74	2.79E-04	6.90E-02
LY6G6D	0.62	2.32	3.74	2.87E-04	6.90E-02
EFHB	1.01	0.24	3.73	2.89E-04	6.90E-02
SORT1	0.75	2.39	3.73	2.92E-04	6.90E-02
BTG1	-1.06	6.24	-3.73	2.96E-04	6.90E-02
MAFA	1.10	-1.92	3.72	2.98E-04	6.90E-02
LIG3	1.17	-0.23	3.72	3.08E-04	6.96E-02
PCDHB11	0.64	3.48	3.71	3.10E-04	6.96E-02
KCNMB3	1.00	1.89	3.70	3.24E-04	6.99E-02
ANO7	0.72	3.47	3.70	3.28E-04	6.99E-02
BLOC1S1	1.10	2.61	3.70	3.29E-04	6.99E-02
QARS1	-0.90	0.50	-3.69	3.36E-04	6.99E-02
FCGR3B	0.96	1.25	3.69	3.39E-04	6.99E-02
PLEC	0.97	1.69	3.68	3.45E-04	6.99E-02
SRSF6	0.93	0.34	3.68	3.47E-04	6.99E-02
NUP214	0.62	2.54	3.67	3.63E-04	6.99E-02
SLCO3A1	0.95	1.33	3.67	3.63E-04	6.99E-02
SH2B3	0.53	4.65	3.67	3.65E-04	6.99E-02
SCLT1	0.48	5.14	3.67	3.67E-04	6.99E-02
MRPL24	1.57	0.28	3.66	3.69E-04	6.99E-02
RNF168	1.55	-2.35	3.66	3.77E-04	7.04E-02
CD48	1.22	1.50	3.65	3.83E-04	7.05E-02
ALAS1	-0.19	6.35	-3.65	3.91E-04	7.08E-02
ANKRD13B	-0.53	12.04	-3.64	3.98E-04	7.08E-02
GRAMD1A	1.21	4.54	3.64	4.00E-04	7.08E-02
RNF227	0.79	1.03	3.64	4.03E-04	7.08E-02
TBX21	0.98	0.65	3.63	4.16E-04	7.12E-02
ZNF439	-0.80	2.16	-3.63	4.21E-04	7.12E-02
LDLRAP1	-0.17	6.57	-3.63	4.23E-04	7.12E-02
RUNX3	0.59	5.13	3.62	4.33E-04	7.12E-02
HDHD2	1.15	3.69	3.62	4.36E-04	7.12E-02
ARSK	1.17	-3.03	3.62	4.38E-04	7.12E-02

MRRF	0.45	7.41	3.61	4.40E-04	7.12E-02
DOK3	-0.27	5.64	-3.61	4.50E-04	7.15E-02
RAP2C	1.22	4.10	3.61	4.53E-04	7.15E-02
PRAMEF2	1.44	0.15	3.60	4.69E-04	7.15E-02
FAM72A	-0.71	2.81	-3.59	4.71E-04	7.15E-02
HIGD1C	0.64	3.04	3.59	4.73E-04	7.15E-02
STAB1	0.63	3.97	3.59	4.74E-04	7.15E-02
CD46	1.48	-0.93	3.59	4.76E-04	7.15E-02
B4GALNT1	-0.30	5.04	-3.59	4.82E-04	7.16E-02
MUTYH	0.22	5.95	3.58	4.90E-04	7.17E-02
ADRB1	-0.54	3.58	-3.58	4.92E-04	7.17E-02
CGRRF1	-0.33	3.49	-3.58	5.01E-04	7.19E-02
CRYAA	0.96	1.01	3.57	5.08E-04	7.19E-02
SMAD1	-0.28	8.35	-3.57	5.09E-04	7.19E-02
BBS7	1.37	-2.91	3.57	5.19E-04	7.26E-02
LYSMD1	-0.48	4.10	-3.56	5.24E-04	7.26E-02
CCDC137	-0.90	-1.73	-3.56	5.30E-04	7.26E-02
MGAT5B	1.17	-0.43	3.56	5.38E-04	7.26E-02
NUDT14	1.18	-1.42	3.55	5.45E-04	7.26E-02
HARS2	-0.78	0.20	-3.55	5.47E-04	7.26E-02
LSM14B	-1.08	-1.69	-3.55	5.48E-04	7.26E-02
ZFAND2B	1.05	1.53	3.54	5.65E-04	7.41E-02
TTF2	0.72	3.53	3.53	5.90E-04	7.67E-02
LILRB1	1.27	3.24	3.52	6.01E-04	7.74E-02
SLC9C2	0.78	2.08	3.52	6.18E-04	7.82E-02
KRTAP2-4	0.83	3.30	3.51	6.33E-04	7.82E-02
MAP4K1	0.72	2.44	3.50	6.42E-04	7.82E-02
ACOX3	-0.34	4.74	-3.50	6.47E-04	7.82E-02
ENC1	0.85	5.49	3.50	6.48E-04	7.82E-02
OCRL	0.87	4.84	3.50	6.53E-04	7.82E-02
ROR1	0.70	3.18	3.50	6.60E-04	7.82E-02
BCL2A1	0.96	2.29	3.49	6.68E-04	7.82E-02
NPAP1	0.80	4.63	3.49	6.69E-04	7.82E-02
DBN1	1.42	3.42	3.49	6.70E-04	7.82E-02
OR6A2	1.12	5.76	3.49	6.70E-04	7.82E-02
MPPED2	-0.76	0.82	-3.49	6.71E-04	7.82E-02
SPATA2	0.93	1.02	3.49	6.76E-04	7.82E-02
TGM2	-0.65	2.88	-3.48	7.06E-04	8.04E-02
CEACAM4	1.45	0.94	3.48	7.08E-04	8.04E-02
NCKAP1L	1.10	4.62	3.47	7.12E-04	8.04E-02
GID8	-0.22	6.29	-3.46	7.35E-04	8.12E-02
TMUB2	-0.32	6.73	-3.46	7.35E-04	8.12E-02
CTRB2	0.77	4.71	3.46	7.37E-04	8.12E-02
ARAP1	-0.64	2.25	-3.46	7.41E-04	8.12E-02
STN1	-0.25	5.30	-3.46	7.55E-04	8.12E-02

SBNO2	0.77	7.20	3.45	7.63E-04	8.12E-02
PPP1R18	1.31	4.39	3.45	7.70E-04	8.12E-02
IL18RAP	0.87	2.87	3.45	7.76E-04	8.12E-02
ZNF202	1.15	1.07	3.45	7.77E-04	8.12E-02
CLHC1	-0.57	6.63	-3.45	7.80E-04	8.12E-02
OR2F1	0.74	1.90	3.45	7.83E-04	8.12E-02
CYTH4	1.27	5.54	3.44	7.86E-04	8.12E-02
SLCO5A1	-0.48	4.89	-3.44	7.91E-04	8.12E-02
CD79A	1.15	1.67	3.43	8.12E-04	8.21E-02
CRNN	0.60	2.34	3.43	8.12E-04	8.21E-02
RNF11	-0.38	4.01	-3.43	8.17E-04	8.21E-02
ANKRD1	-0.65	6.06	-3.43	8.29E-04	8.27E-02
TCEAL9	0.93	7.64	3.41	8.78E-04	8.69E-02
MAP7D3	0.47	4.76	3.41	8.83E-04	8.69E-02
ITIH2	1.10	-1.79	3.41	8.91E-04	8.71E-02
CH25H	-0.62	4.44	-3.40	9.24E-04	8.83E-02
CATSPERD	1.46	1.09	3.39	9.37E-04	8.83E-02
PDE4A	0.34	3.87	3.39	9.43E-04	8.83E-02
ZNF571	0.66	5.20	3.39	9.48E-04	8.83E-02
NAA50	-0.28	6.58	-3.39	9.54E-04	8.83E-02
CLASP2	1.40	-1.47	3.39	9.54E-04	8.83E-02
RPL10L	0.56	2.20	3.39	9.55E-04	8.83E-02
HEBP1	-0.28	3.62	-3.38	9.61E-04	8.83E-02
TCAIM	0.80	2.25	3.38	9.62E-04	8.83E-02
VBP1	1.28	5.20	3.38	9.64E-04	8.83E-02
CYP4F3	0.89	5.72	3.38	9.76E-04	8.88E-02
POC1A	-0.31	5.45	-3.38	9.81E-04	8.88E-02
GOLGA8F	0.86	4.11	3.37	9.96E-04	8.90E-02
PF4	1.03	3.85	3.37	9.98E-04	8.90E-02
IP6K1	1.07	1.47	3.37	1.02E-03	8.90E-02
VSIG2	-0.52	6.84	-3.37	1.02E-03	8.90E-02
CRNKL1	0.27	5.16	3.37	1.02E-03	8.90E-02
GNB2	0.78	4.03	3.37	1.02E-03	8.90E-02
SPNS3	-0.27	4.91	-3.36	1.03E-03	8.90E-02
WTIP	-0.30	3.46	-3.36	1.04E-03	8.90E-02
SAPCD2	-0.32	5.78	-3.36	1.05E-03	8.90E-02
SPATA48	1.24	-0.94	3.36	1.06E-03	8.90E-02
CYP24A1	1.00	2.53	3.36	1.06E-03	8.90E-02
LAPTM4A	-0.25	8.24	-3.36	1.06E-03	8.90E-02
CAMSAP1	0.82	1.78	3.34	1.10E-03	9.16E-02
DIRAS1	-0.33	3.44	-3.34	1.10E-03	9.16E-02
PXDN	0.82	4.85	3.34	1.11E-03	9.16E-02
ARHGEF1	0.58	5.90	3.34	1.12E-03	9.19E-02

SKA1	0.55	3.60	3.34	1.13E-03	9.22E-02
HENMT1	0.87	-1.72	3.33	1.15E-03	9.36E-02
TMEM235	0.47	4.38	3.33	1.16E-03	9.36E-02
SAP30BP	1.04	3.14	3.32	1.18E-03	9.40E-02
CACNA1D	-0.76	0.76	-3.32	1.20E-03	9.40E-02
EMP1	0.90	4.37	3.32	1.20E-03	9.40E-02
SLC30A1	0.51	1.91	3.32	1.20E-03	9.40E-02
SYCN	0.57	1.27	3.32	1.20E-03	9.40E-02
IL32	0.70	4.45	3.32	1.21E-03	9.40E-02
PSMD14	0.62	3.72	3.32	1.21E-03	9.40E-02
CIR1	-0.34	7.41	-3.31	1.21E-03	9.40E-02
FBXO3	-0.24	5.68	-3.31	1.22E-03	9.41E-02
GLB1L	1.22	0.00	3.30	1.25E-03	9.58E-02
NAT10	0.71	5.06	3.30	1.26E-03	9.58E-02
WNK3	-0.54	2.99	-3.30	1.27E-03	9.61E-02
FCHSD2	0.86	1.34	3.30	1.28E-03	9.61E-02
SCUBE2	-0.25	6.61	-3.29	1.30E-03	9.61E-02
CCDC142	1.54	4.43	3.29	1.30E-03	9.61E-02
CHTOP	0.63	2.52	3.29	1.30E-03	9.61E-02
SERP1	1.09	6.61	3.29	1.31E-03	9.61E-02
CD200R1	0.93	0.91	3.29	1.32E-03	9.61E-02
FOXG1	-0.58	6.67	-3.29	1.32E-03	9.61E-02
DMD	-0.47	12.38	-3.29	1.33E-03	9.61E-02
ABI3	0.60	3.12	3.29	1.33E-03	9.61E-02
NRXN1	0.38	5.03	3.28	1.34E-03	9.61E-02
HTN1	-0.72	5.45	-3.28	1.34E-03	9.61E-02
DCAF8	1.14	-0.82	3.28	1.36E-03	9.70E-02
FLT1	1.06	1.93	3.28	1.37E-03	9.70E-02
ZP4	0.55	5.34	3.27	1.38E-03	9.70E-02
SIRPB2	0.35	5.15	3.27	1.38E-03	9.70E-02
EMP2	0.72	2.34	3.27	1.39E-03	9.70E-02
H1-3	0.33	3.91	3.27	1.40E-03	9.75E-02
RHBDD1	-0.28	5.27	-3.27	1.42E-03	9.82E-02
POM121L2	0.33	5.38	3.26	1.45E-03	9.94E-02
TBX20	1.08	-2.29	3.26	1.45E-03	9.94E-02
LPXN	0.51	3.70	3.26	1.45E-03	9.94E-02
PCDHB12	-0.55	2.25	-3.26	1.46E-03	9.96E-02
CROT	-0.40	5.27	-3.25	1.47E-03	9.96E-02

Table S3.

Result of limma DE testing using a sex-adjusted model comparing parents-reported wheezers and controls (adj. P-value < 0.1).
